# Human Gastric Cancer Stem Cell (GCSC) Markers Are Prognostic Factors Correlated With Immune Infiltration of Gastric Cancer

**DOI:** 10.3389/fmolb.2021.626966

**Published:** 2021-05-25

**Authors:** Tong Lin, Wenya Peng, Peipei Mai, E. Zhang, Lisheng Peng

**Affiliations:** ^1^The Fourth Clinical Medical School, Guangzhou University of Chinese Medicine, Shenzhen, China; ^2^Department of Science and Education, Shenzhen Traditional Chinese Medicine Hospital, Shenzhen, China

**Keywords:** gastric cancer, cancer stem cell, marker, prognostic, immune infiltration

## Abstract

The prognosis of patients with gastric cancer (GC) is still unsatisfying. Numerous markers of gastric cancer stem cells (GCSCs) have been identified and were thought to be related to cancer aggressiveness. However, the roles of GCSC markers in GC patients’ prognosis and immune infiltration remain unknown. Expression of GCSC markers was analyzed using Oncomine and Gene Expression Profiling Interactive Analysis (GEPIA). Their associations with clinicopathological parameters were analyzed using UALCAN and LinkedOmics. Alternations and protein expression of GCSC markers were analyzed by cBioPortal and the Human Protein Atlas databases, respectively. The prognostic significance of GCSC markers was evaluated using Kaplan-Meier plotter. Correlations between the expression of GCSC markers and immune infiltration along with biomarkers of tumor-infiltrating immune cells (TIICs) were assessed combined Tumor Immune Estimation Resource and GEPIA. GeneMANIA was used to discover the interactive genes of GCSC markers, and enrichment analysis was performed using Database for Annotation, Visualization, and Integrated Discovery server. We identified six GCSC markers significantly up-expressed in GC, compared with normal stomach tissues. Among them, the overexpression of *ICAM1*, *THY1*, and *CXCR4* significantly indicated adverse, while *EPCAM* indicated beneficial clinicopathological features of GC patients. The up-regulation of *CXCR4* showed unfavorable prognostic significance, whereas *EPCAM* and *TFRC* showed the opposite. The six GCSC markers were all correlated with the infiltration and activation of distinct TIICs. Especially, *ICAM1*, *THY1*, and *CXCR4* showed strongly positive correlations with tumor-associated macrophages. Besides, chemokine, Toll-like receptor, NF-kappa B, and HIF-1 signaling pathways might be involved in the regulation of GCSC markers on cancer development. This study proposed that GCSC markers might be promising targets of GC treatment to weaken cancer stem-like properties and strengthen anticancer immunity.

## Introduction

Gastric cancer (GC) is the fifth most frequently diagnosed and the third leading cause of cancer mortality in the world (Bray et al., 2018). Due to the occult symptoms, over 60% of GC patients are diagnosed at advanced stages where curative surgery is impossible (Thrift and El-Serag, 2020). Chemotherapy and targeted therapy can improve survival and quality of life of unresectable or metastatic GC patients, while drug resistance frequently leads to poor outcomes (Jones and Smyth, 2020; Wei et al., 2020). Over the last 10 years, immunotherapy has revolutionized the field of cancer, especially immune checkpoint inhibitors. However, the clinical efficacy is still unsatisfying in most GC patients (Smyth et al., 2020).

Cancer stem cells (CSCs) are proposed to be a small population of the tumor mass with stem cell properties, which are believed to be responsible for generation, heterogeneity, and sustaining growth of cancer, as well as treatment resistance, recurrence, and metastasis (O’Connor et al., 2014). Abundant studies demonstrated that CSCs share markers with tissue stem cells, and biomarkers of gastric CSCs (GCSCs) have been explored in recent decades (Nguyen et al., 2017). CD44 (*CD44*) was the first identified marker of human GCSC in 2009. After that, epithelial cell adhesion molecules (*EPCAM*), intercellular adhesion molecule 1 (*ICAM1*, also known as CD54), aldehyde dehydrogenase 1 (*ALDH1*), CD90 (*THY1*), CD133 (*PROM1*), transferrin receptor protein 1 (*TFRC*, also known as CD71), signal transducer CD24 (*CD24*), octamer-binding transcription factor 4 (*OCT4*), sex determining region Y-box transcription factor 2 (*SOX2*), leucine-rich repeat-containing G-protein coupled receptor 5 (*LGR5* or *GPR49*), homeobox protein NANOG (*NANOG*), ATP-binding cassette subfamily B member 1 (*ABCB1*), ATP-binding cassette subfamily G member 2 (*ABCG2*), C-X-C motif chemokine receptor 4 (*CXCR4*), CD166 (*ALCAM*), doublecortin-like kinase (*DCLK1*), integrin alpha-6 (*ITGA6*, also known as CD49f), and RNA-binding protein Musashi homolog 1 (*MSI1*) were identified (Ishimoto et al., 2014; Brungs et al., 2016; Bekaii-Saab and El-Rayes, 2017; Nguyen et al., 2017; Fu et al., 2020; Lizárraga-Verdugo et al., 2020). The CSC theory provides a meaningful pointcut that targeting the vital markers or pathways involved in the maintenance of CSCs might influence their stem cell-like properties to eliminate cancer.

Accumulating evidence proved that tumor-infiltrating immune cells (TIICs) in the tumor microenvironment (TME) play critical roles in immune escape and cancer progression, and could serve as prognostic biomarkers. For example, higher infiltrating density of CD4+, CD8+ T cells, and fewer FOXP3+ regulatory T cells (Tregs) could predict improved survival of postsurgical GC patients (Nguyen et al., 2017). Tumor-associated macrophages (TAMs) constitute over half of the immune cells in the TME, and a high density of which are associated with unfavorable outcomes of various cancers (Zhang et al., 2017). Additionally, it was reported that interplays between TIICs and GCSCs might promote cancer (Sainz et al., 2016; Sultan et al., 2017). A better understanding of the interactions between GCSCs and immune infiltration may empower new CSC-directed immunotherapeutic strategies. As yet, the knowledge has not been fully clarified.

In this study, we comprehensively analyzed the expression, alternations, clinicopathological relevance, prognostic value, and correlations with immune infiltration of GCSC markers in GC. Moreover, the interactive genes of GCSC markers were discovered and their biological functions were elucidated to learn the potential regulatory mechanisms of GCSC markers on GC. The findings of our study will uncover the prognostic values of GCSC markers in GC patients, and provide novel insights into the relationships between GCSCs and cancer immunity.

## Materials and Methods

### Analysis of Expression of GCSC Markers in GC

Oncomine^1^ is an online server analyzing microarrays of 18,000 cancer samples integrating data from published literature, the Stanford Microarray Database, and the NCBI Gene Expression Omnibus (GEO) (Rhodes et al., 2007). A meta-analysis of the mRNA expression of GCSC markers in GC and normal stomach tissues was performed by the Oncomine server. The threshold was set as fold change of 1.5, *P*-value of 0.01, and a rank of the top 10% of genes.

Gene Expression Profiling Interactive Analysis (GEPIA)^2^ is a web portal to analyze transcriptome data, based on 9,736 tumors and 8,587 normal samples from the Cancer Genome Atlas (TCGA) and the GTEx projects (Tang et al., 2017). The expression of GCSC markers was further confirmed by GEPIA using GC dataset, with the threshold as *P*-value of 0.01 and fold change of 1.5. The significantly high-expressed GCSC markers overlapping between Oncomine and GEPIA databases were identified using a Venn diagram, which were included in the further investigations.

UALCAN (Chandrashekar et al., 2017)^3^ and LinkedOmics (Vasaikar et al., 2018)^4^ are both interactive platforms for in-depth analysis of cancer omics data from TCGA. Here, these two databases were used to evaluate the expression of GCSC markers in GC patients with distinct clinicopathological parameters, UALCAN was for genders, ages, major stages, and tumor grades; and LinkedOmics was for TNM stages.

### Analysis of Alternations and Protein Expression of GCSC Markers in GC

cBioPortal^5^ is a comprehensive web resource providing visual and multidimensional cancer genomics data (Cerami et al., 2012; Gao et al., 2013). The “TCGA and Firehose Legacy” dataset including data of 478 GC cases was selected in this study. The alternation profiles including mutations, putative copy-number alterations, mRNA expression (z-scores relative to diploid samples with a score threshold of ±2.0), and protein expression (z-scores with a score threshold of ±2.0) were analyzed.

The Human Protein Atlas (HPA) is a program aiming to map all the human proteins in cells, tissues, and organs, the resource of which is openly accessible at https://www.Proteinatlas.org/ (Edfors et al., 2017). The protein expression of GCSC markers in GC tissues and normal stomach tissues were analyzed using immunohistochemistry (IHC) staining data from the HPA. The expression levels of the GCSC markers were quantitated by staining intensity, categorized into negative (none), weak (<25%), medium (25–75%), and strong (>75%).

### Analysis of Prognostic Significance of GCSC Markers in GC Patients

Kaplan-Meier (KM) Plotter^6^ is an online tool for survival analysis of 54 k genes in 21 types of cancers, the data sources of which include the GEO, European Genome-phenome Archive, and TCGA (Györffy et al., 2010). KM Plotter was applied to evaluate the associations between the expression of GCSC markers and overall survival (OS) and relapse-free survival (RFS) of all GC patients, as well as GC patients with distinct clinical parameters. All cases were split into two groups by the median of a gene’s expression.

### Analysis of Correlations Between Expression of GCSC Markers and Immune Infiltration in GC

Tumor Immune Estimation Resource (TIMER)^7^ is a web server for the investigation of tumor-immune interactions, which incorporates 10,897 samples across 32 kinds of cancers from TCGA (Li et al., 2017). The correlations between the expression of GCSC markers and infiltration levels of diverse TIICs, including CD8+ T cells, CD4+ T cells, B cells, neutrophils, macrophages, dendritic cells (DCs), and myeloid-derived suppressor cells (MDSCs) in GC were assessed by TIMER. Furthermore, correlations between expression of GCSC markers and biomarkers of TIICs were studied using GEPIA. Correlation strength was measured by correlation coefficient value referring to the previous studies: 0.00–0.19 is “very weak”, 0.20–0.39 is “weak”, 0.40–0.59 is “moderate”, 0.60–0.79 is “strong”, and 0.80–1.0 is “very strong” (Pan et al., 2019; Xiao et al., 2020).

### Gene Interaction Networks of GCSC Markers and Functional Enrichment Analysis

GeneMANIA^8^ is a web tool for the investigation into associated genes for quired genes through analysis of physical and functional associations, such as co-expression, co-localization, and physical interaction (Franz et al., 2018). We constructed gene interaction networks for every single GCSC marker gene using GeneMANIA. Then, Gene Ontology (GO) and Kyoto Encyclopedia of Genes and Genomes (KEGG) pathway enrichment analysis was performed for the component genes in each gene interaction network, using Database for Annotation, Visualization, and Integrated Discovery (DAVID) server^9^ (Huang et al., 2009). GO enrichment analysis predicted the biological functions of genes in three aspects: biological process (BP), cellular component (CC), and molecular function (MF).

### Statistical Analysis

The comparison of gene expression level in cancer and normal tissues by Oncomine and UALCAN platforms was conducted using student’s *t*-test, that by GEPIA platform was conducted using one-way ANOVA test, and that by LinkedOmics platform was conducted using Kruskal–Wallis test. The association of gene expression and patients’ survival was analyzed using log-rank test, and the survival curve, hazard ratio (HR), 95% confidence interval (CI), and *P*-value were generated. Spearman’s method was applied to evaluate the correlation of gene expression with immune infiltration level or biomarkers’ expression of TIICs. For all the analyses, *P* < 0.05 was considered statistically significant, and a false discovery rate (FDR) <0.05 was an additional criterion for functional enrichment analysis.

## Results

### Expression of GCSC Markers in GC Patients

**FIGURE 1:**
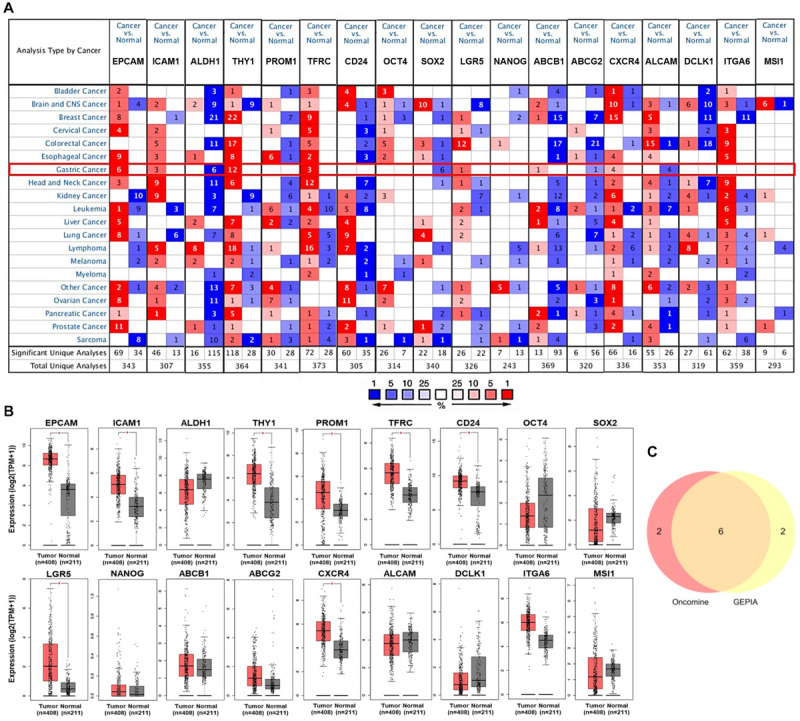
Transcriptional expression of GCSC markers in GC and normal stomach tissues. **(A)** A summary of the datasets in which GCSC markers were significantly up- (red) or down- (blue) expressed in various cancers, compared with the corresponding normal tissues (Oncomine). Numbers in colored cells represent the counts of datasets. The color depth is paralleled with the median rank of a gene across all the included datasets. **(B)** Transcriptional expression of GCSC markers in GC and normal stomach tissues (GEPIA). TPM, transcript per million; *P < 0.01. **(C)** A Venn diagram identified six highly expressed GCSC markers overlapped between Oncomine and GEPIA databases.

Firstly, to determine the differential expression of GCSC markers, the mRNA expression of *EPCAM*, *ICAM1*, *ALDH1*, *THY1*, *PROM1*, *TFRC*, *CD24*, *OCT4*, *SOX2*, *LGR5*, *NANOG*, *ABCB1*, *ABCG2*, *CXCR4*, *ALCAM*, *DCLK1*, *ITGA6*, and *MSI1* in GC and normal stomach tissues were analyzed using the Oncomine and GEPIA databases, respectively. It turned out that eight GCSC markers were significantly higher expressed in GC than normal stomach tissues in Oncomine (Figure 1A) and GEPIA (Figure 1B), respectively. Even though there were some differences in the results from the two databases, the expressions of *EPCAM*, *ICAM1*, *THY1*, *TFRC*, *LGR5*, and *CXCR4* were consistently significantly elevated in both databases, so the six GCSC markers were included in our following study (Figure 1C). As shown in Figure 1A, significant overexpression of the six GCSC markers was observed in a total of 29 GC datasets (Supplementary Table 1). Additionally, they were elevated in numerous datasets of various cancers.

### Expression of GCSC Markers in GC Patients With Distinct Clinicopathological Features

**FIGURE 2:**
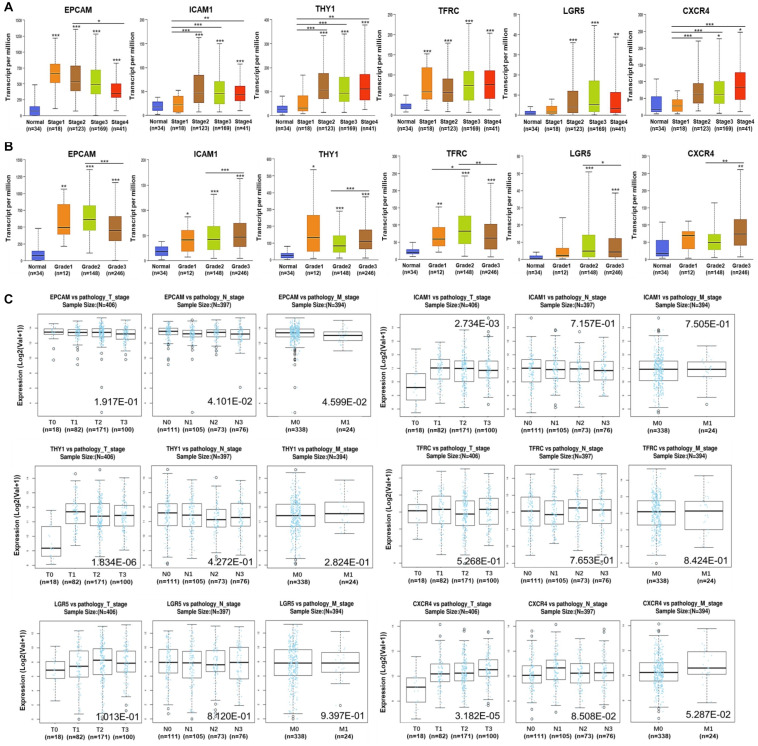
Expression of GCSC markers in GC patients with distinct clinicopathological parameters. Expression of GCSC markers in GC patients with diverse **(A)** pathological stages, **(B)** tumor grades, and **(C)** pathological TNM stages. The box plots and *P-*values in graphs A and B were from UALCAN, while those in graph C were from LinkedOmics; *P < 0.05, **P < 0.01, ***P < 0.001.

Subsequently, the associations of expression of *EPCAM*, *ICAM1*, *THY1*, *TFRC*, *LGR5*, and *CXCR4* with clinicopathological characteristics in GC patients were investigated using UALCAN and LinkedOmics platforms. The expression of the six GCSC markers showed no significant difference in GC patients with distinct genders. In terms of age, *EPCAM* and *TFRC* were up-expressed, whereas *CXCR4* was down-expressed in senile (61–100 years old) patients, compared with that in middle-aged (41–60 years old) patients (*P* < 0.05) (Table 1). Generally, the six GCSC markers were significantly elevated in GC samples with diverse pathological stages and histological grades, compared with normal stomach samples (*P* < 0.05). In particular, *ICAM1*, *THY1*, and *CXCR4* were higher expressed in GC patients in Stage II–IV, compared with that in Stage-I patients (*P* < 0.01) (Figure 2A); and they were expressed higher in Grade-3 tumors than that in Grade-2 tumors (*P* < 0.01) (Figure 2B). In contrast, a declining trend of *EPCAM* expression was observed in patients in advanced stages (Stage II–IV), compared with that in Stage-I patients. Besides, the expression of *EPCAM*, *TFRC*, and *LGR5* was higher in Grade-2 tumors, compared with that in Grade-3 tumors (*P* < 0.05) (Figures 2A,B and Supplementary Table 2).

**TABLE 1:** Expression of GCSC markers in GC patients with distinct genders and ages (UALCAN).

**Gene name**	**EPCAM**		**ICAM1**		**THY1**		**TFRC**		**LGR5**		**CXCR4**	
	**TPM**	***P*-value**	**TPM**	***P*-value**	**TPM**	***P*-value**	**TPM**	***P*-value**	**TPM**	***P*-value**	**TPM**	***P*-value**
**Gender**												
Male (*n* = 268)	517.42	6.51E-01	44.96	7.74E-01	104.98	7.96E-01	68.09	3.54E-01	4.39	7.64E-01	58.22	3.23E-01
Female (*n* = 147)	476.80		45.23		103.83		69.95		4.56		68.72	
**Age**												
21–40 years (*n* = 4)	563.40		76.71		80.09		89.54		0.20		99.11	
41–60 years (*n* = 128)	420.31		39.48		103.72		56.97		2.94		72.93	
61–80 years (*n* = 253)	543.69	**1.66E-06**	48.37	8.64E-01	105.93	1.55E-01	72.20	**3.88E-03**	5.58	5.93E-01	57.86	**9.40E-03**
81–100 years (*n* = 25)	515.85	**1.42E-02**	36.58	1.27E-01	93.64	**2.38E-02**	84.70	7.24E-02	2.94	7.13E-01	56.80	**1.40E-04**

*The expression level of genes was presented with the median of transcript per million (TPM). The *P*-values for 61–80- and 81–100-year-old patients were compared with the 41–60-year-old patients, those of which with statistical significance are in bold. The data of patients in 21–40-year-old was not analyzed due to the insufficient sample size.*

Moreover, the results from LinkedOmics showed elevated expression of *ICAM1* (*P* = 2.73E-03), *THY1* (*P* = 1.83E-06), and *CXCR4* (*P* = 3.18E-05) were significantly associated with advanced T stages (Figure 2C). Overexpression of *EPCAM* was linked with favorable N stages (*P* = 4.101E-02) and M stage (*P* = 4.60E-02). No significant finding of *TFRC* and *LGR5* was identified here. All the above illustrated that the overexpression of *ICAM1*, *THY1*, and *CXCR4* significantly implied adverse outcomes, whereas *EPCAM* implied more optimistic clinicopathological characteristics of GC patients.

### Alternations and Protein Expression of GCSC Markers in GC

**FIGURE 3:**
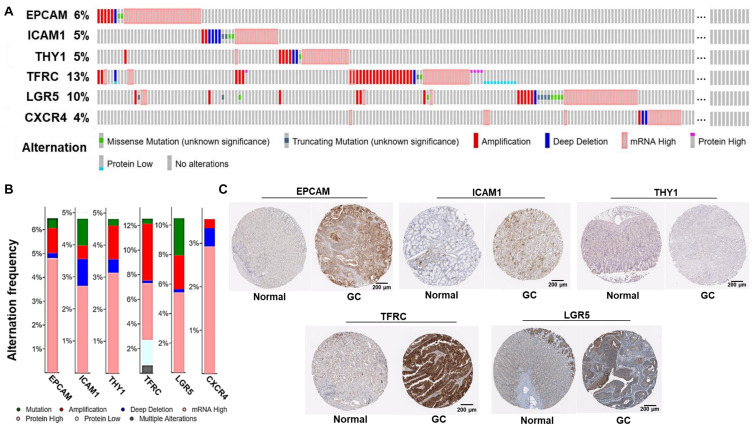
Alternations and protein expression of GCSC markers in GC. **(A)** An overview of the genomic and proteomic alternations of GCSC markers in GC samples from “TCGA, Firehose Legacy” dataset (cBioPortal). **(B)** A summary graph of the alternation frequency of GCSC markers in GC samples (cBioPortal). **(C)** The intensity of IHC staining of GCSC markers in normal stomach and GC tissues (the HPA database). The antibodies for EPCAM, ICAM1, THY1, TFRC, and LGR5 were CAB030012, HPA004877, CAB068244, HPA028598, and HPA012530, respectively. The IHC data of CXCR4 was still in preparation.

Alternations of the GCSC markers in GC patients were analyzed using cBioPortal. In general, seven types of alternations, including missense mutation, truncating mutation, amplification, deep deletion, mRNA overexpression, protein high-expression, and protein low-expression of the GCSC markers were observed in a total of 174 out of 478 (36%) of GC samples; and mRNA overexpression occurred the most frequently. *EPCAM*, *ICAM1*, *THY1*, *TFRC*, *LGR5*, and *CXCR4* were altered in 31 (6.5%), 23 (4.8%), 23 (4.8%), 60 (12.6%), 50 (10.5%), and 17 (3.6%) of all samples, respectively (Figures 3A,B).

Following this, the expression of GCSC marker protein in normal stomach and GC tissues was verified using IHC data from the HPA database, except for CXCR4, whose data was still in preparation. As exhibited in Figure 3C, EPCAM, ICAM1, THY1, TFRC, and LGR5 proteins were strongly, moderately, negatively, strongly, and moderately expressed in GC tissues; which were weakly, negatively, weakly, moderately, and moderately expressed in normal stomach tissues, respectively. In a word, EPCAM, ICAM1, and TFRC proteins were expressed higher in GC than normal stomach tissues, while THY1 and LGR5 proteins were expressed at similar levels in two kinds of tissues.

### Prognostic Significance of GCSC Markers in GC Patients

**FIGURE 4:**
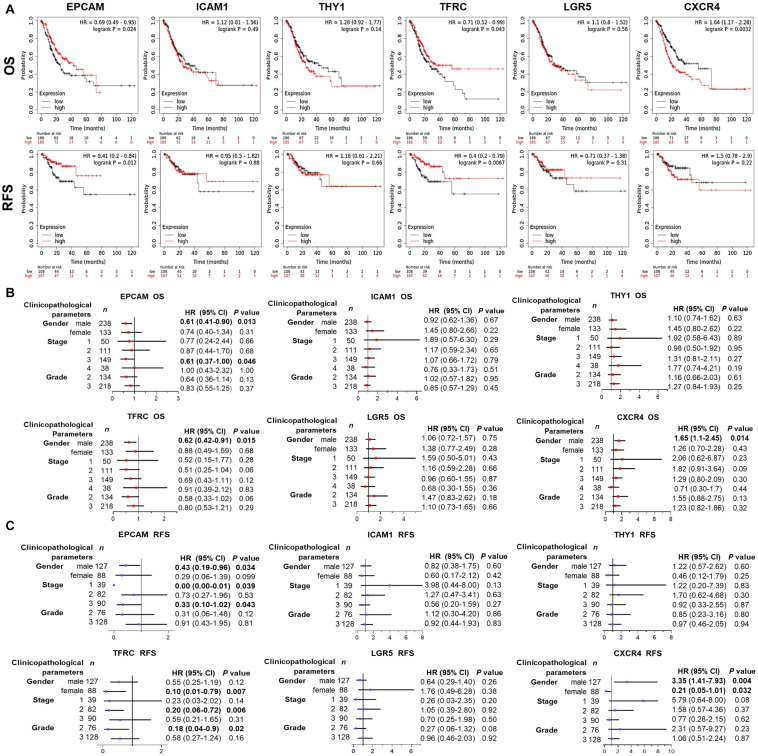
Prognostic significance of GCSC markers in GC patients (KM Plotter). **(A)** The survival curves show the associations between the expression of GCSC markers with OS and RFS of all GC patients. Forest plots show the associations between the expression of GCSC markers with **(B)** OS and **(C)** RFS of GC patients with different clinicopathological parameters. *P-*values with statistical significance are in bold. OS, overall survival; RFS, relapse free survival; HR, hazard ratio; CI, confidence interval.

Wondering whether the expression of GCSC markers affects GC patients’ prognosis, survival analysis was performed using KM Plotter. As shown in Figure 4A, GC patients with high expression of *EPCAM* (OS: HR = 0.69, *P* = 0.024; RFS: HR = 0.41, *P* = 0.012) and *TFRC* (OS: HR = 0.71, *P* = 0.043; RFS: HR = 0.4, *P* = 0.0067) had both better OS and RFS. Nevertheless, high expression of *CXCR4* (OS: HR = 1.64, *P* = 0.0032) was associated with worse survival of GC patients. No significant prognostic indication was found for *ICAM1*, *THY1*, and *LGR5*.

Since the outcomes of cancer patients differ greatly with the clinical parameters, we further assessed the prognostic value of GCSC markers in GC patients with distinct clinicopathological statuses. Concisely, *EPCAM* upregulation indicated both favorable OS and RFS of male GC patients and patients in advanced stage (Stage III) (*P* < 0.05). High-expression of *TFRC* was associated with better OS of male GC patients, and better RFS of GC patients in Stage II, patients with Grade 2 tumors, as well as females (*P* < 0.05). Overexpression of *CXCR4* was correlated with both unfavorable OS and RFS of male patients; paradoxically, it implied better RFS of female patients (*P* < 0.05). Besides, no prognostic significance of *ICAM1*, *THY1*, and *LGR5* was found in this part (Figures 4B,C). Taken together, the findings suggested that up-expressed *EPCAM* and *TFRC* might serve as favorable prognostic indicators, while *CXCR4* might be an unfavorable one for GC patients.

### Correlations Between Expression of GCSCs Markers and Immune Infiltration in GC

**FIGURE 5:**
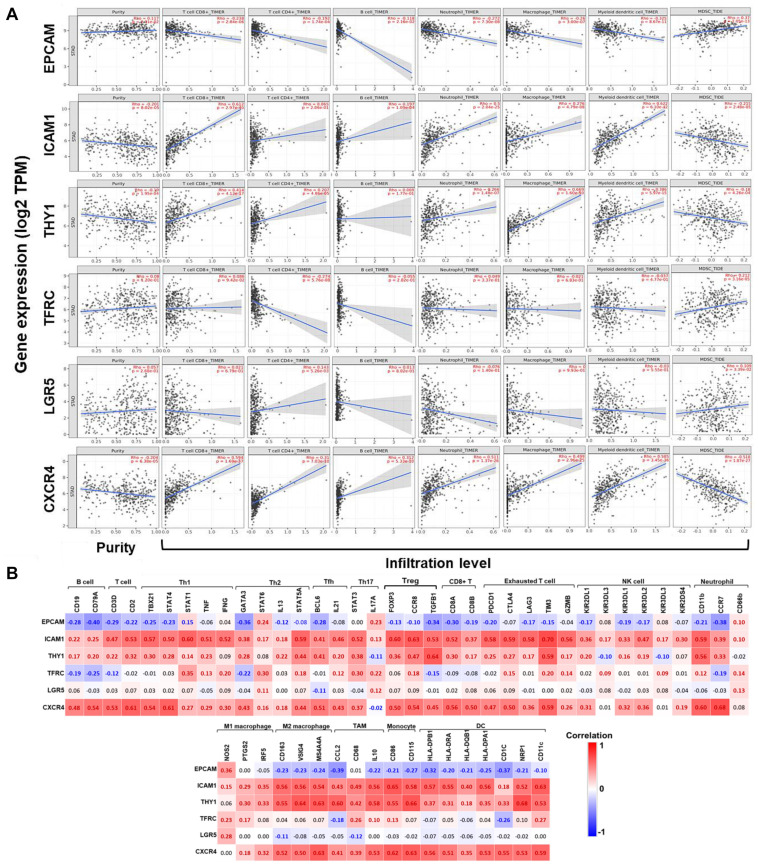
Correlations between the expression of GCSC markers with immune infiltration in GC. **(A)** Correlations between expression of GCSC markers with tumor purity, and infiltration level of CD8+ T cells, CD4+ T cells, B cells, neutrophils, macrophages, DCs, and MDSCs in GC (TIMER). **(B)** Heatmaps show correlations between the expression of GCSC markers with biomarkers of TIICs in GC. Correlation coefficients with statistical significance are in bold, red or blue color represents positive or negative correlation, respectively. TAM, tumor-associated macrophage; Th, helper T cell; Tfh, follicular helper T cell; Treg, regulatory T cell; NK, natural killer cell; DC, dendritic cell; MDSC, myeloid-derived suppressor cell.

Subsequently, correlations between expression of GCSC markers and immune infiltration in GC were investigated by TIMER server. Tumor purity is defined as the proportion of cancer cells in tumor admixture, which can influence the evaluation of immune infiltration. In this study, all the correlation analyses about immune infiltration had been adjusted with the corresponding tumor purity (Aran et al., 2015). It turned out that the expression of *EPCAM* (*P* = 2.31E-02) was positively, while *ICAM1* (*P* = 8.02E-05), *THY1* (*P* = 1.95E-04), and *CXCR4* (*P* = 6.38E-05) were negatively correlated to the tumor purity (Figure 5A), suggesting the expression of *EPCAM* might be mainly from cancer cells, while the latter three genes might be from the cells in the TME.

The expression of *ICAM1*, *THY1*, and *CXCR4* were conformably positively correlated with the infiltration level of CD8+ T cells, CD4+ T cells, B cells, neutrophils, macrophages, and DCs (except for *ICAM1* with CD4+ T cells and *THY1* with B cells), but negatively correlated with that of MDSCs (*P* < 1.10E-04). Interestingly, the correlations between *EPCAM* expression and the infiltration of these TIICs was in an opposite way of *ICAM1*, *THY1*, and *CXCR4* (*P* < 0.05). Furthermore, the expressions of *TFRC* (*P* = 3.16E-05) and *LGR5* (*P* = 3.39E-02) were both positively correlated with the infiltration of MDSCs; but *TFRC* was negatively, while *LGR5* was positively correlated with the infiltration of CD4+ T cells (*P* < 0.01). Notably, the correlation strength of *ICAM1* expression with infiltration of DCs (*r* = 0.622, *P* = 6.10E-42) and CD8+ T cells (*r* = 0.612, *P* = 2.97E-40), *THY1* with macrophages (*r* = 0.669, *P* = 1.60E-50), also *CXCR4* with DCs (*r* = 0.585, *P* = 3.45E-36) were strong.

### Correlations Between the Expression of GCSCs Markers and Biomarkers of TIICs in GC

To further confirm the participation of TIICs, the correlations between the expression of GCSCs markers and biomarkers of TIICs in GC were analyzed using GEPIA. The biomarkers of all TIICs covered in the last step were investigated, along with monocytes, M1/M2 macrophages, subsets of T cells, including helper T cell (Th) 1, Th2, Th17, follicular helper T cell (Tfh), regulatory T cell (Tregs), and exhausted T cells. Consistent with the results from TIMER, the expression of *ICAM1*, *THY1*, and *CXCR4* was positively, while *EPCAM* was negatively correlated with the expression of almost all biomarkers of B cells, T cells, neutrophils, macrophages, monocytes, and DCs (*P* < 0.05) (Figure 5B and Supplementary Table 3). Notably, the expression of *ICAM1*, *THY1*, and *CXCR4* consistently displayed strongly positive correlations with the expression of signatures of M2 macrophages (*CD163*, *VSIG4*, and *MS4A4A*), monocytes (*CD86* and *CD115*), Tregs (*CCR8* and *TGFB1*), exhausted T cells (*TIM3*), neutrophils (*CD11b*), TAM (*IL10*), and DCs (*NRP1* and *CD11c*) (*P* < 9.0E-30). Until now, we could summarize GCSC markers that might modulate immunity through extensive modulation of immune infiltrates in the TME of GC.

### Functions of the Gene Interaction Networks of GCSC Markers

**FIGURE 6:**
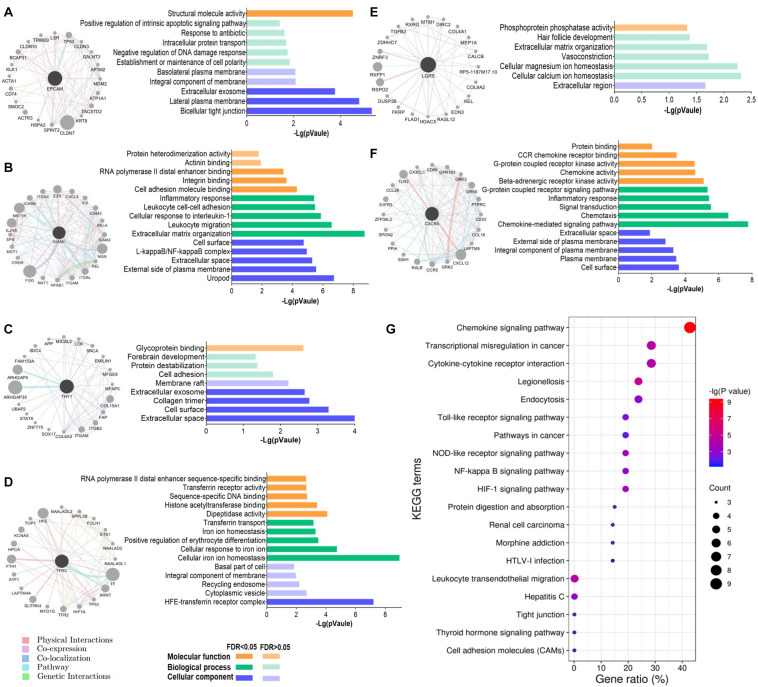
Functions of the interactive genes of GCSC markers. The gene interaction network and its GO functional annotation of **(A)** *EPCAM*, **(B)** *ICAM1*, **(C)** *THY1*, **(D)** *TFRC*, **(E)** *LGR5*, and **(F)** *CXCR4*. Distinct colors of the edges in gene interaction networks represent distinct interactions, including physical interactions, co-expression, co-localization, pathway, and genetic interactions (GeneMANIA). **(G)** All the significantly enriched KEGG pathway terms of the six gene interaction networks are shown. The size of a bubble indicates the number of genes enriched for a pathway term; a redder color of a bubble represents a greater significance of the corresponding term.

To understand the biological functions of these GCSC markers, a gene interaction network was constructed for each gene using GeneMANIA. Twenty interactive genes were identified for each GCSC marker gene; thus, a gene interaction network was composed of a total of 21 genes (Figures 6A–F). Subsequently, functional enrichment analysis was performed for all constituent genes in each network using DAVID platform. The five most significantly enriched or all GO-CC, GO-BP, and GO-MF terms were shown as bar plots. It was uncovered that the interactive genes of *EPCAM*, *ICAM1*, *THY1*, *LGR5*, and *CXCR4* were all components of the external cell membrane. Beyond that, *EPCAM*’s interactive genes participated in the maintenance of cell polarity, negative regulation of DNA damage response, protein transport, and positive regulation of apoptosis (Figure 6A). *ICAM1*’s and *THY1*’s interactive genes were both responsible for cell adhesion, and *ICAM1*’s interactive genes also partook in the cellular response to interleukin-1 (IL-1) and binding of RNA polymerase II and integrin (Figures 6B,C). *TFRC*’s interactive genes were components of transferrin receptor complex and majored in iron ion homeostasis (Figure 6D). *LGR5*’s interactive genes were involved with cellular ions homeostasis (Figure 6E), while *CXCR4*’s interactive genes regulated chemokine-mediated inflammatory response and G-protein coupled receptor kinase activity (Figure 6F).

Furthermore, all the significantly enriched KEGG pathway terms of the total six gene interaction networks were presented in Figure 6G, which expounded that they were related to the processes of transcriptional disorder in cancer, cytokine-cytokine receptor interaction, cell adhesion, etc. Specifically, signaling pathways of chemokine, Toll-like receptor, cancer, nucleotide-binding oligomerization domain (NOD)-like receptor, nuclear factor (NF)-kappa B, and hypoxia inducible factor-1 (HIF-1) were involved.

## Discussion

Despite dramatic progress in diagnosis and treatment, the prognosis of GC patients remains poor, primarily blamed on the frequent treatment resistance, following relapse and metastasis. The CSCs are believed to contribute to the inefficacy of conventional therapies, for their quiescent nature, capabilities of anti-apoptosis, DNA damage repair, and diminished uptake and/or enhanced efflux of drugs (Zhao, 2015). In this scenario, eliminating CSCs should be an effective strategy to eradicate cancer, otherwise, recurrence might be unavoidable (Sainz et al., 2016). Up to date, most studies were conducted in cell lines or mice, reports describing roles of GCSC markers in GC patients were limited (Fu et al., 2018). In this study, the ever-reported GCSC markers were comprehensively analyzed in aspects of expression, alternations, prognostic significance, as well as their interactions with immune infiltration in GC patients.

From the start, six GCSC markers, *EPCAM*, *ICAM1*, *THY1*, *TFRC*, *LGR5*, and *CXCR4* were identified significantly high-expressed and frequently altered in GC, compared with normal stomach samples. Among them, we that found upregulation of *ICAM1*, *THY1*, and *CXCR4* was associated with pathological and histological advancement of GC patients, but only *CXCR4* showed unfavorable prognostic significance. Consistent with the previous studies, *CXCR4* had been widely reported as an unfavorable prognostic indicator of various cancers, including GC. *CXCR4* was elevated in GC and was closely associated with cancer progression and metastasis (Xiang et al., 2017; Jiang et al., 2019). Surprisingly, we found that *CXCR4* over-expression indicated better RFS of female GC patients, which needs further explorations with larger sample sizes. *ICAM1* was ever identified as an unfavorable prognostic biomarker in earlier studies (Jung et al., 2012; Chen et al., 2020). High *THY1* expression was reported to relate with aggressive clinicopathological features of GC cases (Numakura et al., 2019), and the silencing of which would inhibit the malignant traits and enhance apoptosis of GC cells (Wu et al., 2019). However, we found that *ICAM1* and *THY1* had no prognostic significance.

The previous studies demonstrated that *EPCAM* overexpression was an independent unfavorable prognostic factor and was linked with larger tumor size and lymph node metastasis in GC patients (Chen et al., 2016; Dai et al., 2017). Unexpectedly, we found that *EPCAM* upregulation suggested more optimistic clinicopathological characteristics and outcomes of GC patients, even for the advanced-stage patients. In fact, the beneficial role of *EPCAM* in prognosis had been observed in ovarian cancer (Woopen et al., 2014) and pancreatic cancer (Meng et al., 2015) before. Besides, *TFRC* high-expression implied better histological differentiation status and favorable prognosis of GC patients. Our findings could be supported by an earlier study that *TFRC*—cells represented high tumorigenicity, multipotency, invasiveness, and treatment resistance (Ohkuma et al., 2012). Even though it was demonstrated that *LGR5* overexpression might contribute to the progressive clinical features and poor OS of GC patients (Zheng et al., 2013; Chen et al., 2016; Huang et al., 2016), we had no similar findings in this study. To summarize, our findings highlighted that the up-expression of *CXCR4* as unfavorable, whereas *EPCAM* and *TFRC* as favorable prognostic biomarkers of GC patients. Some findings inconsistent with the previous studies might lie in the intrinsic heterogeneity of cancer and different quantification methods. Therefore, more investigations with larger sample sizes are still required.

After that, we observed that the expression of GCSC markers was correlated with the infiltration and biomarkers expression of TIICs in the TME of GC. High expression of *ICAM1*, *THY1*, and *CXCR4* was positively, while *EPCAM* was negatively correlated with the infiltration level and biomarkers expression of T cells, B cells, neutrophils, macrophages, and DCs. In particular, the expression of *ICAM1*, *THY1*, and *CXCR4* exhibited strongly positive correlations with the biomarkers of M2 TAMs, TAM, monocytes, Tregs, and DCs. Furthermore, the expression of *TFRC* and *LGR5* was positively, whereas *ICAM1*, *THY1*, and *CXCR4* were negatively correlated with the infiltration of MDSCs.

It is acknowledged that CD8+ T cells are the main undertakers of anticancer immunity, a high infiltration level of which indicates better treatment response and prognosis (Liu et al., 2015; Solinas et al., 2017). On the contrary, TAMs, Tregs, and MDSCs are the major immunosuppressive constituents in the TME. TAMs can be polarized into M1- and M2- types (Solinas et al., 2017); M1 TAMs enhance immunity surveillance by producing proinflammatory cytokines; while M2 TAMs operate oppositely by producing anti-inflammatory cytokines (Gambardella et al., 2020). A high abundance of M2 TAMs was usually associated with worse OS in various cancers, including GC (Yin et al., 2017), and the conversion of M2- to M1- type might induce tumor regression (Hagemann et al., 2008). Additionally, emerging evidence indicated M2 TAMs could foster cancer stemness in various cancers (Raggi et al., 2016; Zhang et al., 2017). The increased number of Tregs, especially the FOXP3+ ones, has been applied as an independent risk factor of cancer relapse for their roles in immunity tolerance (Rezalotfi et al., 2019). In a recent study, co-culture with Tregs might increase *LGR5* expression in GC cells, which conferred poor prognosis (Liu X. S. et al., 2019). Evidence proved that GCSCs could affect the balance of Th17/Treg, which was considered a promising diagnostic indicator of CSCs’ activation (Rezalotfi et al., 2019). MDSCs can dampen the proliferation and function of T cells via secreting inhibitory molecules or depriving their essential nutrient substances (Kumar et al., 2016). Furthermore, MDSCs can induce the generation of Tregs and TAMs to promote CSC properties synergistically (Ugel et al., 2015; Peng et al., 2016; Ruiu et al., 2019). DCs are antigen-presenting cells specialized in T cells trigger, however, subpopulations of DCs can act both immune- stimulatory and suppressive in cancer, depending on the secretion of inflammatory cytokines (Volovitz et al., 2016). A study announced that plasmacytoid DCs in peripheral blood and tumor tissue forecasted poor outcomes of GC patients (Liu X. et al., 2019). Whereas, another study indicated DCs loaded with mRNA of CSC-like cells from GC patients might stimulate effective immune responses (Bagheri et al., 2019). Hereto, we could conclude that GCSC markers would affect the clinicopathological features and even prognosis of GC patients by directly affecting cancer cells and modulating the immune microenvironment. We speculated that high-expression of *ICAM1*, *THY1*, and *CXCR4* might incline to promote immunosuppression, whereas *EPCAM* and *TFRC* tended to enhance immunity surveillance. The interplay between CSCs and the immune microenvironment is a promising breakthrough point for cancer therapy. Targeting CSCs can curtail cancer aggressiveness, and it is an attractive strategy to adjust anticancer immunity at the same time.

At last, the results of GO functional annotation revealed that *EPCAM*’s interactive genes were responsible for cell polarity and regulation of DNA damage response. The interactive genes of *ICAM1* and *THY1* were both involved in cell adhesion, and the former also regulated cellular response to cytokine and mRNA translation. *TFRC*’s and *LGR5*’s interaction networks consistently partook in cellular ions homeostasis. And *CXCR4* mainly acted as a chemokine-mediated inflammatory regulator. The KEGG enrichment analyses elucidated that signal pathways of chemokine, Toll-like receptor, cancer, NOD-like receptor, NF-kappa B, and HIF-1 were involved. Toll-like receptor, NOD-like receptor, and NF-kappa B pathways all play vital roles in cancer-related inflammation, which process proinflammatory cytokines engaging in cancer progression (Hu et al., 2016; Cao and Xu, 2019). HIF-1 signaling affects cell proliferation, apoptosis, angiogenesis, epithelial-mesenchymal transition, and drug resistance of diverse cancer cells, including GC (Li et al., 2019), and it could also facilitate CSCs generation and cancer aggressive phenotype (Das et al., 2008).

In conclusion, we found that elevated *CXCR4* might serve as an unfavorable, while *EPCAM* and *TFRC* might be favorable prognostic biomarkers of GC patients. The GCSC markers might affect the disease outcomes by maintaining the stem cell-like properties of cancer and remodeling distinct TIICs in the TME, especially TAMs, Tregs, and DCs. Specifically, the processes of cell adhesion, mRNA translation, and inflammation, and signaling pathways of chemokine, Toll-like receptor, NF-kappa B, and HIF-1 might be involved. Our study suggested that GCSC markers were promising therapeutic targets to diminish cancer aggressivity and modulate anticancer immunity in GC. Despite this, there is still a long way to go for experimental and clinical validations.

## Data Availability Statement

All the data that support the findings of this study have been provided in the article/Supplementary Material, or are publicly available in the databases mentioned in the Materials and Methods section.

## Author Contributions

TL designed the study, conducted bioinformatics analysis, and wrote the original manuscript. WP and PM studied the relevant literature. EZ refined the figures. LP provided comments and guidance of the manuscript. All authors have read, contributed to the revision of the manuscript, and approved the submitted version.

## Conflict of Interest

The authors declare that the research was conducted in the absence of any commercial or financial relationships that could be construed as a potential conflict of interest.

## Footnotes

1 https://www.oncomine.org/resource/login.html

2 http://gepia.cancer-pku.cn

3 http://ualcan.path.uab.edu

4 http://www.linkedomics.org/

5 http://www.cbioportal.org/

6 http://www.kmplot.com/

7 http://timer.cistrome.org

8 http://genemania.org

9 https://david.ncifcrf.gov/home.jsp
