# Supplementary material for: Human Gastric Cancer Stem Cell (GCSC) Markers Are Prognostic Factors Correlated With Immune Infiltration of Gastric Cancer
Source: Front Mol Biosci. 2021 May 25;8:626966. doi: 10.3389/fmolb.2021.626966 (PMC8185345; doi:10.3389/fmolb.2021.626966)
Supplement: Supplementary file 1 [file Table_1.pdf]

SUPPLEMENTARY TABLE 1 The significantly differential expression of GCSC markers between GC and normal stomach tissues (Oncomine)

Gene name	Types of GC vs. normal stomach	Fold change	*P* value	t-Test	Reference
EPCAM	Gastric mixed adenocarcinoma vs. normal	3.65	3.91E-07	6.40	Cho Gastric (1)
	Gastric intestinal type adenocarcinoma vs. normal	2.57	6.17E-54	4.32	
	Diffuse gastric adenocarcinoma vs normal	2.32	1.11E-04	4.05	
	Gastric cancer vs. normal	1.94	6.56E-04	3.67	Wang Gastric (2)
	Gastric cancer vs. normal	1.66	3.64E-04	3.45	Cui Gastric (3)
	Gastric intestinal type adenocarcinoma vs. normal	2.15	2.99E-07	5.77	DErrico Gastric (4)
ICAM1	Diffuse gastric adenocarcinoma vs normal	2.65	1.00E-03	4.03	DErrico Gastric (4)
	Gastric intestinal type adenocarcinoma vs. normal	3.06	3.11E-08	6.30	
	Gastric cancer vs. normal	1.95	2.00E-03	3.20	Wang Gastric (2)
THY1	Diffuse gastric adenocarcinoma vs. normal	6.25	5.98E-14	14.71	Chen Gastric (5)
	Gastric intestinal type adenocarcinoma vs. normal	2.75	3.46E-21	13.35	
	Gastric mixed adenocarcinoma vs. normal	6.90	5.67E-06	8.55	
	Gastric intestinal type adenocarcinoma vs. normal	4.89	7.60E-11	9.04	Cho Gastric (6)
	Diffuse gastric adenocarcinoma vs. normal	4.80	1.61E-12	9.24	
	Gastric mixed adenocarcinoma vs. normal	3.13	1.91E-04	4.75	
	Gastric adenocarcinoma vs. normal	4.39	7.00E-03	4.51	
	Gastric cancer vs. normal	4.70	9.25E-06	5.57	Wang Gastric (2)
	Gastric cancer vs. normal	2.86	1.57E-05	4.29	Cui Gastric (7)
	Gastric intestinal type adenocarcinoma vs. normal	2.78	3.92E-10	8.10	DErrico Gastric (4)
	Diffuse gastric adenocarcinoma vs. normal	3.00	2.00E-03	4.88	
	Gastric mixed adenocarcinoma vs. normal	3.69	5.17E-04	8.37	
TFRC	Gastric mixed adenocarcinoma vs. normal	2.07	1.16E-06	6.18	Cho Gastric (6)
	Diffuse gastric adenocarcinoma vs. normal	1.70	5.05E-06	5.09	
	Gastric cancer vs. normal	1.81	1.00E-03	3.43	Wang Gastric (2)
LGR5	Gastric cancer vs. normal	2.76	5.33E-05	3.98	Cui Gastric (7)
CXCR4	Gastric mixed adenocarcinoma ys. normal	2.03	8.67E-04	3.95	Chen Gastric (5)
	Diffuse gastric adenocarcinoma vs. normal	1.83	9.96E-04	3.50	
	Gastric cancer vs. normal	2.29	1.00E-03	3.40	Wang Gastric (2)
	Diffuse gastric adenocarcinoma vs. normal	2.82	3.00E-03	4.11	DErrico Gastric (4)

Reference:

1. Cho JY, Lim JY, Cheong JH, Park YY, Yoon SL, Kim SM et al. Gene expression signature-based prognostic risk score in gastric cancer. *Clin Cancer Res*. (2011) 7:1850-7. doi: 10.1158/1078-0432.CCR-10-2180

2. Wang Q, Wen YG, Li DP, Xia J, Zhou CZ, Yan DW et al. Upregulated INHBA expression is associated with poor survival in gastric cancer. *Med Oncol*. (2012) 1:77-83. doi: 10.1007/s12032-010-9766-y

3. Cui J, Chen Y, Chou WC, Sun L, Chen L, Suo J et al. An integrated transcriptomic and computational analysis for biomarker identification in gastric cancer. *Nucleic Acids Res*. (2011) 4:1197-207. doi: 10.1093/nar/gkq960

4. D'Errico M, de Rinaldis E, Blasi MF, Viti V, Falchetti M, Calcagnile A et al. Genome-wide expression profile of sporadic gastric cancers with microsatellite instability. *Eur J Cancer*. (2009) 3:461-9. doi: 10.1016/j.ejca.2008.10.032

5. Chen X, Leung SY, Yuen ST, Chu KM, Ji J, Li R, et al. Variation in gene expression patterns in human gastric cancers. *Mol Biol of Cell*. (2003) 8:3208-15. doi: 10.1091/mbc.e02-12-0833

6. Cho JY, Lim JY, Cheong JH, Park YY, Yoon SL, Kim SM, et al. Gene expression signature-based prognostic risk score in gastric cancer. *Clin Cancer Res*. (2011) 7:1850-7. doi: 10.1158/1078-0432.CCR-10-2180

7. Cui J, Chen Y, Chou WC, Sun L, Chen L, Suo J, et al. An integrated transcriptomic and computational analysis for biomarker identification in gastric cancer. *Nucleic Acids Res*. (2011) 4:1197-207. doi: 10.1093/nar/gkq960
