# Supplementary material for: Human Gastric Cancer Stem Cell (GCSC) Markers Are Prognostic Factors Correlated With Immune Infiltration of Gastric Cancer
Source: Front Mol Biosci. 2021 May 25;8:626966. doi: 10.3389/fmolb.2021.626966 (PMC8185345; doi:10.3389/fmolb.2021.626966)
Supplement: Supplementary file 2 [file Table_2.pdf]

SUPPLEMENTARY TABLE 2 Comparison of GCSC markers' expression in GC patients with different pathological stages and histological grades

Gene name	EPCAM *P* value	ICAM1 *P* value	THY1 *P* value	TFRC *P* value	LGR5 *P* value	CXCR4 *P* value
Normal vs. Stage1	**4.00E-05**	2.46E-01	1.02E-01	**2.42E-04**	**9.44E-02**	8.19E-02
Normal vs. Stage2	**4.09E-09**	**1.76E-09**	**<1E-12**	**<1E-12**	**9.19E-10**	6.42E-02
Normal vs. Stage3	**2.93E-08**	**3.98E-12**	**1.62E-12**	**1.62E-12**	**1.54E-09**	**2.27E-02**
Normal vs. Stage4	**6.84E-04**	**3.68E-05**	**1.18E-06**	**1.87E-08**	**2.56E-03**	**2.19E-02**
Stage1 vs. Stage2	7.08E-01	**3.99E-05**	**4.72E-08**	8.49E-01	5.94E-01	**3.51E-09**
Stage1 vs. Stage3	3.92E-01	**5.60E-05**	**1.18E-06**	4.49E-01	9.71E-01	**3.63E-12**
Stage1 vs. Stage4	9.79E-02	**5.22E-03**	**1.01E-04**	2.99E-01	7.16E-01	**1.43E-04**
Stage2 vs. Stage3	3.21E-01	5.10E-01	1.59E-01	2.22E-01	8.74E-02	4.88E-01
Stage2 vs. Stage4	**4.42E-02**	1.85E-01	6.23E-01	1.89E-01	7.44E-01	2.39E-01
Stage3 vs. Stage4	1.60E-01	3.82E-01	2.09E-01	6.41E-01	4.00E-01	3.97E-01
Normal vs. Grade 1	**1.26E-03**	**4.57E-02**	**1.36E-02**	**2.82E-03**	1.16E-01	4.57E-01
Normal vs. Grade 2	**1.71E-12**	**2.82E-11**	**4.44E-16**	**1.62E-12**	**1.34E-10**	2.63E-01
Normal vs. Grade 3	**4.27E-07**	**<1E-12**	**1.62E-12**	**1.62E-12**	**1.68E-12**	**7.04E-03**
Grade 1 vs. Grade 2	3.22E-01	8.04E-01	1.65E-01	**4.54E-02**	6.70E-01	9.66E-01
Grade 1 vs. Grade 3	0.00E+00	0.00E+00	0.00E+00	0.00E+00	0.00E+00	0.00E+00
Grade 2 vs. Grade 3	**2.51E-06**	**9.68E-05**	**1.68E-05**	**3.80E-03**	**1.51E-02**	**2.34E-03**

Note: The comparison was conducted using student's t-test. The *P* values with statistical significance are in bold.
