# Supplementary material for: Human Gastric Cancer Stem Cell (GCSC) Markers Are Prognostic Factors Correlated With Immune Infiltration of Gastric Cancer
Source: Front Mol Biosci. 2021 May 25;8:626966. doi: 10.3389/fmolb.2021.626966 (PMC8185345; doi:10.3389/fmolb.2021.626966)
Supplement: Supplementary file 3 [file Table_3.pdf]

SUPPLEMENTARY TABLE 3 Correlations between the expression of GCSC markers and biomarkers of TIICs in GC (GEPIA)

**Types of TIICs**	**Gene markers**	**EpCAM**		**ICAM1**		**THY1**		**TFRC**		**LGR5**		**CXCR4**	
		r	***P***	***r***	***P***	***r***	***P***	***r***	***P***	***r***	***P***	***r***	***P***
B cell	CD19	-0.280	**1.6E−08**	0.220	**1.2E−05**	0.170	**7.90E-04**	−0.19	**8.7E−05**	0.058	2.40E-01	0.480	**4.6E−25**
	CD79A	−0.4	**1.1E−16**	0.250	**3E−07**	0.200	**4.7E−05**	−0.25	**2.8E−07**	−0.028	5.70E-01	0.540	**1.1E−32**
T cell (general)	CD3D	−0.29	**2.8E−09**	0.470	**1.9E−23**	0.220	**8.1E−06**	−0.12	**1.80E-02**	0.030	5.40E-01	0.530	**2.6E−31**
	CD2	−0.22	**6.7E−06**	0.530	**1.2E−30**	0.320	**1.7E−11**	−0.015	7.60E-01	0.066	1.80E-01	0.610	**2.9E−43**
Th1	TBX21	−0.25	**2.2E−07**	0.570	**1.9E−36**	0.300	**1.2E−09**	−0.012	8.00E-01	0.028	5.70E-01	0.540	**8.3E−33**
	STAT4	−0.23	**2.1E−06**	0.500	**1.2E−26**	0.280	**1.5E−08**	0.029	5.60E-01	0.020	6.90E-01	0.610	**3E−42**
	STAT1	0.150	**3.00E-03**	0.600	**9.5E−42**	0.140	**3.80E-03**	0.350	**2.3E−13**	0.074	1.30E-01	0.270	**2.8E−08**
	TNF	−0.06	2.30E-01	0.510	**3E−28**	0.230	**3.6E−06**	0.130	**7.90E-03**	−0.046	3.50E-01	0.290	**1.7E−09**
	IFNG	0.036	4.70E-01	0.520	**3.1E−30**	0.091	6.70E-02	0.200	**3.2E−05**	0.086	8.40E-02	0.300	**4E−10**
Th2	GATA3	−0.36	**3.6E−14**	0.380	**1.2E−15**	0.280	**4.8E−09**	−0.22	**9.3E−06**	−0.043	3.80E-01	0.430	**1.1E−19**
	STAT6	0.240	**1.2E−06**	0.170	**7.60E-04**	0.077	1.20E-01	0.300	**1.1E−09**	0.110	**2.10E-02**	0.160	**1.50E-03**
	IL13	−0.12	**1.70E-02**	0.180	**2.10E-04**	0.220	**1E−05**	0.028	5.70E-01	0.001	9.80E-01	0.180	**3.80E-04**
	STAT5A	−0.075	1.30E-01	0.590	**4.4E−39**	0.440	**1.3E−20**	0.180	**2.50E-04**	0.073	1.40E-01	0.440	**2.8E−20**
Tfh	BCL6	−0.28	**6.1E−09**	0.410	**3.4E−18**	0.410	**4.1E−18**	−0.0084	8.70E-01	−0.11	**2.40E-02**	0.510	**3.1E−28**
	IL21	−0.079	1.10E-01	0.460	**1.2E−22**	0.200	**6E−05**	0.120	**1.70E-02**	0.029	5.70E-01	0.430	**1.8E−19**
Th17	STAT3	0.004	9.40E-01	0.520	**2.5E−29**	0.380	**1.6E−15**	0.300	**7.5E−10**	−0.042	4.00E-01	0.370	**9.2E−15**
	IL17A	0.230	**3.8E−06**	0.130	**1.10E-02**	−0.11	**2.90E-02**	0.220	**6.5E−06**	0.120	**1.40E-02**	−0.017	7.40E-01
Treg	FOXP3	−0.13	**1.00E-02**	0.600	**7.1E−42**	0.360	**3.6E−14**	0.061	2.20E-01	0.071	1.50E-01	0.500	**1.7E−27**
	CCR8	−0.098	**4.70E-02**	0.630	**2E−47**	0.470	**8.4E−24**	0.180	**3.40E-04**	0.086	8.10E-02	0.540	**3.1E−32**
	TGFB1	−0.34	**2.7E−12**	0.530	**2.4E−30**	0.640	**3E−49**	−0.15	**2.10E-03**	−0.0076	8.80E-01	0.450	**1E−21**
CD8+ T	CD8A	−0.3	**1.2E−09**	0.520	**5.2E−30**	0.300	**7.3E−10**	−0.087	7.90E-02	0.015	7.60E-01	0.560	**9.6E−35**
	CD8B	−0.19	**1E−04**	0.370	**2.6E−14**	0.170	**4.30E-04**	−0.079	1.10E-01	0.075	1.30E-01	0.500	**2.4E−27**
T cell exhaustion	PDCD1	−0.2	**6.4E−05**	0.580	**3.7E−38**	0.250	**3.6E−07**	−0.016	7.50E-01	0.059	2.30E-01	0.470	**9.6E−24**
	CTLA4	−0.065	1.90E-01	0.590	**8.8E−40**	0.270	**2.4E−08**	0.150	**1.80E-03**	0.093	6.00E-02	0.500	**1E−26**
	LAG3	−0.17	**7.10E-04**	0.580	**1.5E−37**	0.170	**4.80E-04**	0.014	7.90E-01	−0.011	8.20E-01	0.360	**3.4E−14**
	TIM3	−0.15	**2.10E-03**	0.700	**1.8E−60**	0.590	**4.8E−39**	0.200	**7E−05**	−0.0038	9.40E-01	0.590	**1E−39**
	GZMB	−0.042	3.90E-01	0.560	**2.8E−35**	0.170	**7.90E-04**	0.140	**4.40E-03**	0.018	7.20E-01	0.260	**8.7E−08**
NK cell	KIR2DL1	−0.17	**5.30E-04**	0.360	**2.8E−14**	0.200	**5.7E−05**	0.019	7.00E-01	−0.031	5.30E-01	0.310	**1.8E−10**
	KIR3DL3	0.079	1.10E-01	0.170	**5.60E-04**	−0.099	**4.70E-02**	0.092	6.30E-02	0.081	1.00E-01	0.007	8.90E-01
	KIR3DL1	−0.19	**8.6E−05**	0.330	**7.8E−12**	0.160	**9.90E-04**	0.014	7.80E-01	−0.024	6.20E-01	0.320	**6.2E−11**
	KIR3DL2	−0.17	**7.10E-04**	0.470	**1.8E−23**	0.190	**1E−04**	0.012	8.00E-01	0.033	5.00E-01	0.360	**4.2E−14**
	KIR3DL3	0.079	1.10E-01	0.170	**5.60E-04**	−0.099	**4.70E-02**	0.092	6.30E-02	0.081	1.00E-01	0.007	8.90E-01
	KIR2DS4	−0.071	1.50E-01	0.300	**3.3E−10**	0.067	1.80E-01	0.012	8.10E-01	−0.039	4.30E-01	0.190	**1E−04**
Neutrophil	CD11b	−0.21	**3E−05**	0.590	**6.1E−40**	0.560	**1.1E−34**	0.120	**1.40E-02**	−0.061	2.20E-01	0.600	**1.2E−41**
	CCR7	−0.38	**3.5E−15**	0.390	**1.1E−16**	0.330	**4.8E−12**	−0.19	**1.70E-04**	−0.034	5.00E-01	0.680	**1.3E−56**
	CD66b	0.100	**3.80E-02**	0.100	**3.50E-02**	−0.023	6.50E-01	0.140	**5.60E-03**	0.130	**7.40E-03**	0.078	1.10E-01
M1 Macrophage	NOS2	0.360	**9.3E−14**	0.150	**2.70E-03**	0.059	2.30E-01	0.230	**2E−06**	0.280	**1.5E−08**	0.001	9.90E-01
	PTGS2	0.003	9.60E-01	0.290	**2.3E−09**	0.300	**5.3E−10**	0.170	**7.10E-04**	−0.0012	9.80E-01	0.180	**3.40E-04**
	IRF5	−0.048	3.40E-01	0.350	**2.1E−13**	0.330	**7E−12**	0.077	1.20E-01	0.003	9.60E-01	0.320	**4.2E−11**
M2 Macrophage	CD163	−0.23	**2E−06**	0.560	**1.9E−34**	0.550	**5.9E−33**	0.040	4.30E-01	−0.11	**3.00E-02**	0.520	**1.8E−29**
	VSIG4	−0.23	**3.8E−06**	0.560	**4.2E−35**	0.640	**1.7E−48**	0.061	2.20E-01	−0.083	9.20E-02	0.500	**3.8E−27**
	MS4A4A	−0.24	**1.1E−06**	0.540	**6.9E−33**	0.630	**1.1E−46**	0.072	1.50E-01	−0.051	3.00E-01	0.630	**7.3E−46**
TAM	CCL2	−0.39	**3.9E−16**	0.430	**4.8E−2**	0.600	**1.7E−41**	−0.18	**2.80E-04**	−0.048	3.30E-01	0.410	**4.4E−18**
	CD68	0.007	8.80E-01	0.490	**1.1E−25**	0.420	**3.6E−19**	0.260	**1.5E−07**	−0.12	**1.90E-02**	0.390	**2.3E−16**
	IL10	−0.22	**8E−06**	0.560	**9.6E−35**	0.580	**1.3E−37**	0.098	**4.70E-02**	−0.00087	5.60E-01	0.530	**2.4E−31**
Monocyte	CD86	−0.21	**3E−05**	0.650	**1.5E−49**	0.550	**3.2E−33**	0.130	**8.00E-03**	−0.028	5.70E-01	0.620	**3.4E−45**
	CD115	−0.27	**2.6E−08**	0.580	**2.5E−37**	0.660	**4.5E−52**	0.067	1.80E-01	−0.023	6.50E-01	0.630	**4.3E−47**

DC	HLA-DPB1	−0.32	**4.9E−11**	0.570	**5.3E−36**	0.370	**4.7E−15**	−0.07	1.60E-01	−0.072	1.50E-01	0.560	**1.7E−35**
	HLA-DRA	−0.2	**4.1E−05**	0.550	**3.3E−33**	0.310	**1E−10**	0.053	2.80E-01	−0.049	3.30E-01	0.510	**5.6E−29**
	HLA-DQB1	−0.21	**1.6E−05**	0.400	**6.4E−17**	0.180	**3.10E-04**	−0.059	2.30E-01	−0.06	2.20E-01	0.350	**1.5E−13**
	HLA-DPA1	−0.25	**2.1E−07**	0.560	**4.7E−35**	0.350	**4.2E−13**	0.036	4.70E-01	−0.047	3.40E-01	0.530	**1.5E−30**
	CD1C	−0.37	**5.2E−15**	0.180	**2E−04**	0.330	**1.6E−11**	−0.26	**7.2E−08**	−0.02	6.90E-01	0.550	**2.4E−33**
	NRP1	−0.21	**2.3E−05**	0.520	**4.5E−29**	0.680	**1.6E−56**	0.097	5.00E-02	−0.019	7.00E-01	0.530	**1.8E−30**
	CD11c	−0.1	**3.60E-02**	0.630	**4.2E−47**	0.530	**1.2E−30**	0.270	**2.7E−08**	−0.0014	9.80E-01	0.590	**1.4E−39**

Note: The correlation analysis had been adjusted for the tumor purity. *P* values with statistical significance are in bold. TIICs, tumor infiltrating immune cells; r, correlation coefficient of Spearman’s analysis; Th: helper T cell; Tfh: follicular helper T cell; Treg, regulatory T cell; NK cell, natural killer cell; TAM: tumor-associated macrophage; DC, dendritic cells.
